# Androgen Receptor Promotes Ligand-Independent Prostate Cancer Progression through c-Myc Upregulation

**DOI:** 10.1371/journal.pone.0063563

**Published:** 2013-05-21

**Authors:** Lina Gao, Jacob Schwartzman, Angela Gibbs, Robert Lisac, Richard Kleinschmidt, Beth Wilmot, Daniel Bottomly, Ilsa Coleman, Peter Nelson, Shannon McWeeney, Joshi Alumkal

**Affiliations:** 1 Division of Hematology/Oncology, Knight Cancer Institute, Oregon Health and Science University, Portland, Oregon, United States of America; 2 Oregon Clinical and Translational Research Institute, Portland, Oregon, United States of America; 3 Department of Medical Informatics and Clinical Epidemiology; Division of Bioinformatics and Computational Biology; Oregon Health and Science University, Portland, Oregon, United States of America; 4 Fred Hutchinson Cancer Research Center, University of Washington, Seattle, Washington, United States of America; University of Kentucky College of Medicine, United States of America

## Abstract

The androgen receptor (AR) is the principal therapeutic target in prostate cancer. For the past 70 years, androgen deprivation therapy (ADT) has been the major therapeutic focus. However, some patients do not benefit, and those tumors that do initially respond to ADT eventually progress. One recently described mechanism of such an effect is growth and survival-promoting effects of the AR that are exerted independently of the AR ligands, testosterone and dihydrotestosterone. However, specific ligand-independent AR target genes that account for this effect were not well characterized. We show here that *c-Myc,* which is a key mediator of ligand-independent prostate cancer growth, is a key ligand-independent AR target gene. Using microarray analysis, we found that *c-Myc* and AR expression levels strongly correlated with each other in tumors from patients with castration-resistant prostate cancer (CRPC) progressing despite ADT. We confirmed that AR directly regulates *c-Myc* transcription in a ligand-independent manner, that *AR* and *c-Myc* suppression reduces ligand-independent prostate cancer cell growth, and that ectopic expression of c-Myc attenuates the anti-growth effects of AR suppression. Importantly, treatment with the bromodomain inhibitor JQ1 suppressed c-Myc function and suppressed ligand-independent prostate cancer cell survival. Our results define a new link between two critical proteins in prostate cancer – AR and c-Myc – and demonstrate the potential of AR and c-Myc-directed therapies to improve prostate cancer control.

## Introduction

Prostate cancer is the most common cancer in men in the United States with 241,740 new cases anticipated this year [1]. Despite screening and early treatment, prostate cancer commonly recurs, and 28,170 men are predicted to die from prostate cancer this year [1]. Nearly all of these prostate cancer deaths are attributable to metastatic, castration-resistant prostate cancer (CRPC) that has progressed despite androgen deprivation therapy (ADT) – the most common treatment for patients with recurrent or advanced prostate cancer.

ADT works by lowering levels of the potent AR ligands testosterone and dihydrotestosterone (DHT) or interfering with binding of androgen ligands to the androgen receptor (AR) protein, the principal therapeutic target in prostate cancer [2]. Despite ADT, including novel and more potent treatments, all prostate cancers eventually progress [3], [4]. At progression, the AR is ubiquitously expressed [5], [6].

There are several possible explanations for AR-dependent mechanisms of progression despite the suppression or interference with androgen ligands. These include intratumoral androgen synthesis, the generation of constitutively active AR transcript variants, AR gene amplification, activating AR mutations, or activation of the AR by growth factors [7]–[16]. It is also now clear that the AR protein can promote the activation of AR ligand-independent pathways distinct from AR’s canonical ligand-activated pathways in CRPC [17]. However, critical downstream AR target genes of this type that account for AR dependent, ligand-independent prostate cancer cell survival have not been fully clarified. The study of such AR target genes and mechanisms by which AR regulates their expression will improve our understanding of castration-resistance and lead to the identification of key AR dependent proteins whose activity may control growth and survival of CRPC cells. Such targets and pathways would naturally become high priorities for drug development.

To understand genes that might account for that effect, we focused on *c-Myc*. This is because: 1) *c-Myc* overexpression promotes prostate cancer development [18]; 2) *c-Myc* is upregulated in androgen ligand-dependent prostate cancer and further upregulated in CRPC [19], [20]; and 3) prior reports have demonstrated that c-Myc, like AR, contributes to ligand-independent prostate cancer cell growth [21]. Our review of prior data that localized AR binding sites throughout the genome by chromatin immunoprecipitation (ChIP) showed that the AR localizes to an enhancer element of the *c-Myc* gene [17]. However, it was unclear if *c-Myc* was a direct AR target gene and whether androgen ligands were necessary for AR regulation of *c-Myc* expression.

We determined that *c-Myc* upregulation in human CRPC tumors correlates with *AR* upregulation, and we confirmed that *c-Myc* is a direct AR target gene using chromatin immunoprecipitation (ChIP) assays. *c-Myc* suppression achieves the same overall effects as *AR* suppression, and *c-Myc* overexpression attenuates the anti-growth effects of *AR* suppression. While AR promotes *c-Myc* expression, treatment with androgen ligands did not increase *c-Myc* expression. Thus, AR promotes the expression of *c-Myc* in a ligand-independent manner, and *c-Myc* is a key AR target gene.

Finally, we treated prostate cancer cells with the BET bromodomain inhibitor JQ1 that suppresses c-Myc expression [22], [23]. Treatment with JQ1 achieved the same overall effect as *c-Myc* RNAi and reduced prostate cancer cell survival in androgen ligand-depleted conditions.

Our studies clarify that *c-Myc* is a key androgen ligand-independent AR target gene that contributes to androgen ligand-independent but AR-dependent prostate cancer cell survival. Our results also demonstrate the potential of AR-directed therapies or c-Myc-directed therapies in prostate cancer as adjuncts to ADT.

## Results

### AR and *c-Myc* are Concordantly Expressed in Metastatic CRPC

**Figure 1:**
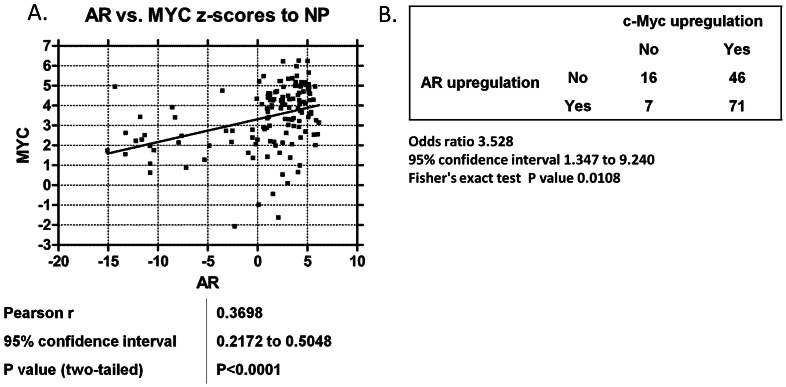
*AR* and *c-Myc* levels are positively correlated in CRPC specimens. A) Z-scores (to normal prostate specimens) of *AR* versus *c-Myc* mRNA expression across 140 human CRPC metastases. The Pearson correlation coefficient, linear regression, and F test for significantly non-zero slope were performed for each pair of genes. B) Fisher’s exact test and odds ratio on the contingency table analyzing the co-occurrence of tumors with *AR* or *c-Myc* z-scores greater than 2.

Both AR and c-Myc are critical survival pathways in prostate cancer, and expression levels of both *AR* and *c-Myc* are commonly increased in human CRPC tumors progressing despite ADT [24], [25]. However, it was unknown whether overexpression of *AR* and *c-Myc* was linked with the other in human CRPC tumors. Therefore, we determined the expression levels of *AR* and *c-Myc* using gene expression microarrays in 140 human CRPC tumors versus 15 normal prostate samples. Next, we examined the association of *AR* upregulation and *c-Myc* upregulation in the human CRPC tumors. *AR* mRNA levels in CRPC samples were strongly associated with *c-Myc* mRNA levels (Pearson correlation = 0.3698, 95% Confidence Interval: 0.2172–0.5048, two-tailed p-value<0.0001) (Figure 1A). We also calculated the odds ratio for *c-Myc* and *AR* upregulation in these CRPC specimens. There was a statistically significant association with *AR* upregulation and *c-Myc* upregulation (OR = 3.528, 95% Confidence Interval: 1.347 to 9.240, p-value: 0.0108 by Fisher's Exact Test) (Figure 1B).

### AR Suppression Reduces the Growth of AR Ligand-dependent and AR Ligand–independent Castration-resistant Prostate Cancer Cells

**Figure 2:**
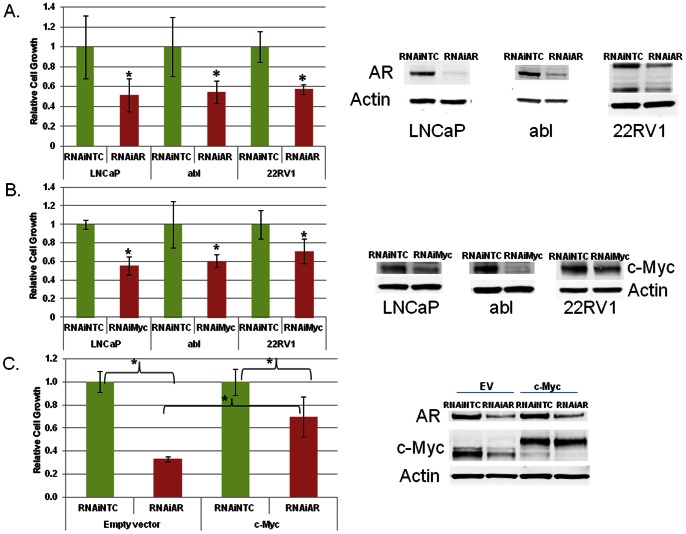
AR and c-Myc promote ligand-independent prostate cancer cell growth. A) LNCaP, abl and 22RV1 cells were transfected with 50 nM of non-targeted control (NTC) or *AR* RNAi oligonucleotides. Cells were switched to charcoal-stripped serum on the day of transfection. Cell growth was determined 5 days later for LNCaP and 6 days later for abl and CRPC 22RV1 with the trypan blue exclusion method. B) Immunoblot for AR expression. The lower bands in the AR immunoblot in 22RV1 cells reflect the presence of an AR transcript variant [7]. B) LNCaP, abl and 22RV1 cells were transfected with 50 nM of NTC or *c-Myc* RNAi oligonucleotides. Cells were switched to charcoal-stripped serum on the day of transfection. Cell growth was determined 5 days later for LNCaP cells and 6 days later for abl and 22RV1 with the trypan blue exclusion method. Immunoblot for c-Myc protein expression. C) LNCaP cells with stable overexpression of empty vector (EV) or c-Myc were generated. These cells were transfected with 50 nM of non-targeted control (NTC) or AR siRNA oligonucleotides. Cell growth was determined 6 days later with the trypan blue exclusion method. Immunoblot for AR and c-Myc protein expression. The higher bands on the c-Myc immunoblot in the c-Myc-overexpressing cells represent the ectopically-expressed c-Myc. *denotes p<0.05 compared to NTC.

We suppressed expression of the *AR* with RNAi in prostate cancer cells grown in charcoal-stripped, androgen ligand-depleted serum. *AR* RNAi reduced cell growth of both androgen ligand-dependent LNCaP cells and their CRPC derivatives called LNCaP-abl (Figure 2A). Of note, both of these cells only express the full-length AR transcript. *AR* suppression with RNAi in the 22RV1 CRPC cell line that expresses both full-length AR and an AR transcript variant achieved the same effect (Figure 2A). In all cell lines, AR suppression reduced cell growth without inducing apoptosis (data not shown), suggesting a defect in proliferation. Thus, despite androgen ligand depletion, *AR* suppression further reduces prostate cancer cell growth.

### c-Myc Suppression Recapitulates the Effect of AR Suppression, and c-Myc Overexpression Attenuates the Anti-tumor Activity of AR Suppression

To determine if c-Myc also influenced prostate cancer cell growth independent of androgen ligands, we suppressed *c-Myc* using RNAi. Like *AR* downregulation, *c-Myc* down-regulation suppressed ligand-independent growth of LNCaP, abl, and 22RV1 cells (Figure 2B). Further, we simultaneously suppressed *AR* and *c-Myc* with RNAi. Co-suppression of both proteins did not reduce cell growth more than suppression of either *AR* or *c-Myc* by itself (Figure S1).

We also demonstrated that *c-Myc* overexpression conferred ligand-independent growth to ligand-dependent LNCaP cells propagated long-term in castrate conditions, which is concordant with a prior report (Figure S2) [21]. Next, we suppressed *AR* with RNAi in LNCaP cells overexpressing empty vector or *c-Myc* and quantified cell growth. *c-Myc* overexpression was protective against the growth suppressive effects of *AR* RNAi (Figure 2C). This demonstrates that *c-Myc* at least partially contributes to AR’s effects on promoting ligand-independent prostate cancer cell survival.

### AR but not Androgens Promote c-Myc Expression

The *c-Myc* oncogene is commonly upregulated in prostate cancer, and *c-Myc* upregulation promotes ligand-independent prostate cancer cell survival [21]. However, the dependency of *c-Myc* expression on AR had not been established. Accordingly, we examined previously published ChIP microarray data that localized AR throughout the genome of androgen ligand-dependent LNCaP cells and their Abl CRPC derivatives [17]. AR was reported to be bound to an enhancer element of the *c-Myc* gene in both of these cell lines. Therefore, we next used ChIP to confirm these results.

**Figure 3:**
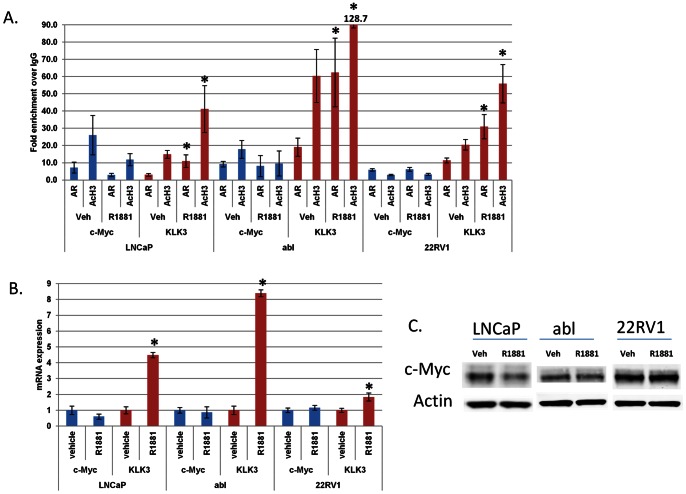
*c-Myc* expression is not activated by androgen ligands. LNCaP, abl, and 22RV1 cells were grown in charcoal-stripped serum for 72 hours and then treated with 10 nM R1881 (or ethanol vehicle) for 4 hours. A) Chromatin immunoprecipitation was performed to determine the enrichment of AR and histone H3 acetylation (AcH3) at the *c-Myc* and *KLK3* enhancer elements. B) QRT-PCR was performed to determine the mRNA levels of *KLK3* and *c-Myc* relative to actin. C) Immunoblotting was performed to determine the protein levels of AR, c-Myc, and actin. *denotes p<0.05 compared to vehicle.

First, we grew cells in charcoal-stripped, androgen ligand-depleted serum and determined the effect of treatment with the androgen ligand R1881 on AR occupancy and histone acetylation, a mark of active transcription, at the *c-Myc* enhancer element using ChIP. We also measured the effects of R1881 treatment at the well-described, ligand-activated gene *KLK3*. AR and high levels of histone acetylation were present at the *c-Myc* enhancer even when cells were grown in ligand-depleted serum (Figure 3A). Further, the addition of R1881 to culture did not enhance AR occupancy or histone acetylation at *c-Myc* (Figure 3A). This contrasts with the effect of R1881 at the *KLK3* gene enhancer- increased enrichment of AR and histone acetylation (Figure 3A).

We next determined the effect of R1881 treatment on expression of *c-Myc* or *KLK3*. R1881 treatment increased *KLK3* expression (Figure 3B). However, R1881 treatment did not increase *c-Myc* expression. Figure 3B,C).

Next, we treated prostate cancer cells with MDV3100, a potent, new androgen antagonist [26]. MDV3100 treatment suppressed expression of *KLK3* but did not affect expression of *c-Myc*. (Figure S3). These results further support the notion that androgen ligands do not promote expression of *c-Myc*.

**Figure 4:**
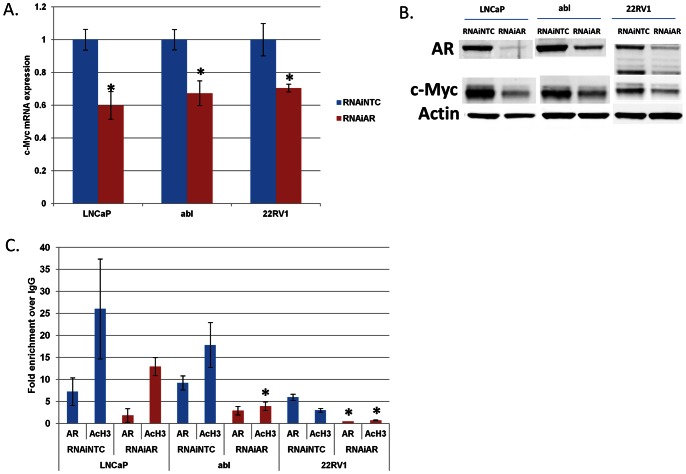
AR promotes ligand-independent expression of *c-Myc*. LNCaP, abl and 22RV1 cells were transfected with 50 nM of non-targeted control (NTC) or AR RNAi oligonucleotides. Cells were then grown in charcoal-stripped serum for 96 hours. At the end of the treatment, cells were harvested to extract mRNA and protein. A) QRT-PCR was performed to determine the levels of *c-Myc* relative to *actin*. B) Immunoblotting was performed to determine the levels of AR, c-Myc and actin. C) Parallel treatments were performed and cells were cross-linked and processed for ChIP to determine AR and histone H3 acetylation (AcH3) enrichment at the *c-Myc* enhancer. *denotes p<0.05 compared to NTC.

To determine if the AR was capable of regulating *c-Myc* in a ligand-independent manner, we used RNAi to suppress the expression of *AR* and measured *c-Myc* expression. RNAi-mediated suppression of *AR* reduced c-Myc mRNA and protein expression (Figure 4A, B). We performed ChIP assays and confirmed that *AR* RNAi reduced AR and histone acetylation from the *c-Myc* enhancer (Figure 4C). This was most significant in the 22RV1 cell line, although strong trends were also seen in LNCaP and Abl cells. Thus, *c-Myc* is a direct AR target gene, and *AR* RNAi suppresses *c-Myc* expression at least in part through depletion of AR and histone acetylation from the *c-Myc* enhancer. We also overexpressed AR in the M12 prostate cancer cell line that does not normally express AR. AR overexpression increased c-Myc mRNA and protein expression (Figure S4). This further supports the notion that AR activates *c-Myc* expression in a ligand-independent manner.

### AR Suppression Recapitulates the Effect of c-Myc Suppression

**Figure 5:**
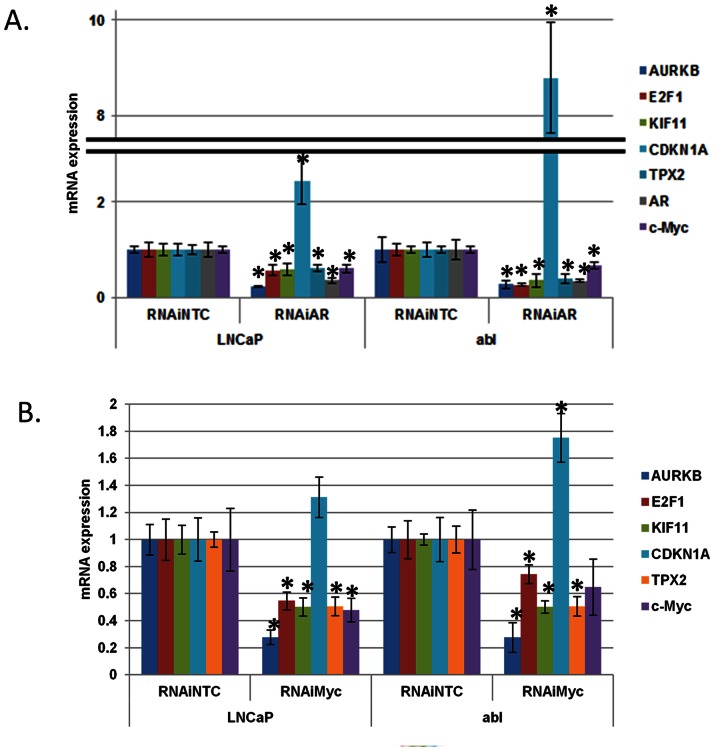
*AR* suppression recapitulates the effect of *c-Myc* suppression on *c-Myc* target gene expression. LNCaP and abl cells were transfected with 50 nM of non-targeted control (NTC) and either A) *AR* or B) *c-Myc* RNAi oligonucleotides. Cells were switched to charcoal-stripped serum on the day of transfection and harvested 96 hours later. QRT-PCR was performed to determine the levels of the indicated *c-Myc* target genes relative to *actin*. *denotes p<0.05 compared to NTC.

We next determined whether RNAi-mediated suppression of *AR* recapitulated the effect of RNAi-mediated suppression of *c-Myc* on expression of well-described *c-Myc* target genes (Figure 5) [27], [28]. Both *c-Myc* RNAi and *AR RNAi* reduced expression of the *c-Myc*-activated gene *E2F1*; conversely, *c-Myc* and *AR* RNAi both increased expression of the *c-Myc*-repressed gene *CDKN1A* (Figure 5). Recent reports demonstrate that mitotic genes, including *KIF11*, *AURKB*, and *TPX2*, are key c-Myc target genes [29]–[31]. *AR* RNAi recapitulated the effect of c*-Myc* RNAi and also reduced expression of these genes (Figure 5). This demonstrates that AR suppression disrupts c-Myc function and expression of well-established c-Myc target genes.

### The BET Bromodomain Inhibitor JQ1 Suppresses c-Myc Function and Reduces AR Ligand-independent Prostate Cancer Cell Survival

**Figure 6:**
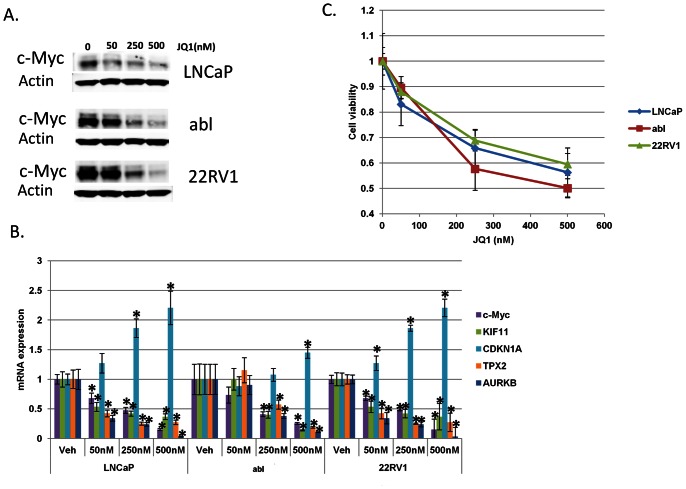
JQ1 treatment suppresses c-Myc expression and function and reduces ligand-independent prostate cancer cell survival. LNCaP, abl, and 22RV1 cells were grown in charcoal-stripped serum and treated with vehicle, 50 nM, 250 nM or 500 nM JQ1 every 24 hours for 72 hours. A) Immunoblotting was performed to determine the protein levels of c-Myc. B) QRT-PCR was performed to determine the mRNA level of *c-Myc* and c-Myc targets genes *KIF11, CDKN1A, TPX2*, and *AURKB* relative to *actin*. *denotes p<0.05 compared to vehicle. C) Cell viability was determined at the end of treatment with the trypan blue exclusion method. p<0.01 for the 250 nM and 500 nM doses vs. vehicle in all three cell lines.

Our results demonstrate that *c-Myc* is an important AR target gene but that *c-Myc*’s expression is not activated by androgenic ligands. Currently, therapies to suppress AR expression are not yet available. However, recent work demonstrates that a BET bromodomain inhibitor called JQ1 suppresses c-Myc expression and c-Myc function because *c-Myc* is a bromodomain target gene [22], [23]. Therefore, we treated prostate cancer cells with JQ1. JQ1 treatment reduced mRNA and protein levels of c-Myc (Figure 6A,B) and suppressed c-Myc function as measured by c-Myc target gene expression (Figure 6B). Finally, like *c-Myc* RNAi, JQ1 treatment with nanomolar concentrations reduced ligand-independent prostate cancer cell survival (Figure 6C).

## Discussion

It is well-appreciated that the AR is a critical driver of prostate cancer cell survival and that AR accounts for progression to fatal CRPC despite treatment with ADT [24]. In many cases androgens persist intracellularly within CRPC tumors despite castrate serum levels of androgens [24], [32]. However, androgen ligand-independent but AR-dependent mechanisms that also promote survival of CRPC cells have been reported. These include activation of the AR by IL-6, AR gene amplification, and AR transcript variants that lack the androgen ligand binding domain [7]–[9], [15]. All of these mechanisms may contribute to prostate cancer progression despite ADT since none are directly targeted by ADT.

Recently, it was demonstrated that the AR protein promotes the expression of a gene program distinct from its canonical androgen ligand-directed targets in CRPC cells [17]. One such example is the AR target gene *UBE2C* that promotes ligand-independent prostate cancer proliferation [17]. Which of the other AR-induced gene products is critical for ligand-independent prostate cancer cell survival has been unclear. Our work demonstrates that the *c-Myc* oncogene is such a ligand-independent AR target gene.

*c-Myc* is commonly upregulated in prostate cancer, and *c-Myc* overexpression transforms normal prostatic epithelial cells in genetically engineered mouse models of prostate cancer and confers ligand-independent prostate cancer cell survival, but the dependency of *c-Myc* expression on the AR was unclear [18], [20], [21], [33]. We show here that *AR* and *c-Myc* are commonly upregulated in CRPC, and we confirmed that *AR* and *c-Myc* upregulation strongly correlated with each other in a large series of metastatic CRPC patient tumors (Figure 1).

We confirmed that *AR* suppression leads to loss of c-Myc expression in prostate cancer cell lines expressing full-length AR (LNCaP and Abl) and in another CRPC cell line 22RV1 that expresses both full-length AR and an AR transcript variant. Although we cannot exclude a role for the AR transcript variant in also promoting *c-Myc* expression, our results with AR RNAi in LNCaP and Abl and our results with AR overexpression in M12 cells demonstrate that full-length AR is capable of activating *c-Myc* expression (Figure 4, Figure S4).

Like *AR* RNAi, *c-Myc* RNAi reduced prostate cancer cell survival in androgen ligand-depleted conditions while co-suppression of *AR* and *c-Myc* was not more effective than suppression of either protein alone (Figure 2, Figure S1). *c-Myc* overexpression confers ligand-independent survival to prostate cancer cells (Figure S2), which matches a prior report [21]. We also showed that *c-Myc* overexpression attenuated the anti-tumor activity of *AR* suppression with RNAi (Figure 2C). Thus, c-Myc contributes to AR’s effects on promoting ligand-independent prostate cancer cell survival.

Despite the fact that AR promotes expression of *c-Myc*, treatment with androgen ligand did not increase *c-Myc* expression (Figure 3). Additionally, treatment with androgen ligands did not enhance AR occupancy at the *c-Myc* enhancer (Figure 3). This contrasts with the effects of androgen stimulation on expression of the *KLK3* gene, a well-described androgen-activated gene, and AR occupancy at the *KLK3* enhancer (Figure 3). To further confirm that AR promotes expression of *c-Myc* in a ligand-independent manner, we treated prostate cancer cells with the new, potent androgen antagonist MDV3100 [26]. Treatment with MDV3100 in a recent randomized, placebo-controlled phase III clinical trial improved overall survival of patients with CRPC [34]. However, in some patients there is no tumor response, and at progression the AR remains in the nucleus [3], [6], [34]. While MDV3100 treatment reduced expression of *KLK3*, MDV3100 treatment had no effect on *c-Myc* expression (Figure S3). These data further support the notion that AR promotes *c-Myc* expression in a ligand-independent manner. *c-Myc* is a critical factor in ligand-independent prostate cancer progression (Figure 2, Figure S2) [21]. Therefore, in the future, it will be important to measure *c-Myc* expression and function in CRPC patient tumors progressing despite more complete androgen interference with drugs such as MDV3100.

Because of the importance of *c-Myc* as a downstream contributor to AR’s effects on ligand-independent prostate cancer cell survival, we treated prostate cancer cells with the BET bromodomain inhibitor JQ1, a drug known to suppress expression of bromodomain target genes; foremost among which was *c-Myc* [22], [23]. In prior studies, JQ1 treatment in vitro and in vivo suppressed c-Myc expression and function and suppressed tumor growth without appreciable toxicity [22], [23].

We confirmed that JQ1 treatment of prostate cancer cells using nanomolar concentrations achieved the same overall effects as RNAi-mediated suppression of *c-Myc* – c-Myc mRNA and protein depletion, suppression of c-Myc function, and suppression of ligand-independent prostate cancer cell survival (Figure 6). In light of the involvement of *c-Myc* in critical physiological processes, targeting the c-Myc protein generally in multiple cell types through long-term administration could be undesirable [35], [36]. Clinical trials will be necessary to determine the safety of bromodomain inhibition. However, the results to date, including our own, suggest that this is a promising anti-tumor strategy for the treatment of CRPC (Figure 6) [22], [23].

**Figure 7:**
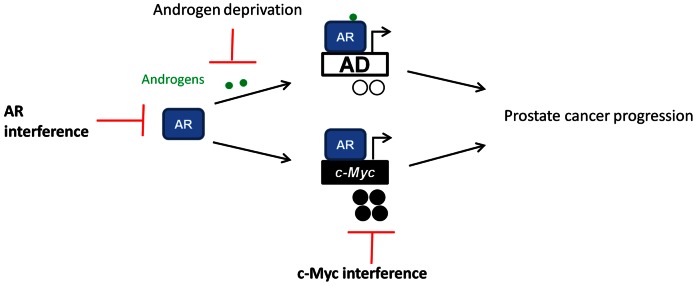
AR and c-Myc are critical drivers of ligand-independent mechanisms of prostate cancer progression. Currently, androgen deprivation therapies that interfere with androgen ligand activation of the AR are primarily used to treat this disease. These therapies suppress AR’s androgen ligand-dependent function and suppress expression of androgen ligand-dependent (AD) AR target genes. However, despite these treatments prostate cancer progression is inevitable. The AR also promotes the expression of androgen ligand-independent pathways such as *c-Myc*. The *c-Myc* gene is commonly upregulated in prostate cancer and contributes to androgen ligand-independent prostate cancer progression. This model strongly suggests that AR or c-Myc-directed therapies would complement current androgen deprivation strategies.

Finally, that the AR controls expression of its target genes such as *c-Myc* in a tissue specific manner suggests that the ideal agent for suppression of *c-Myc* expression in prostate cancer cells specifically would target AR, itself. Indeed, our studies clarify that androgen ligand-independent but AR-dependent *c-Myc* gene upregulation is a mechanism by which the AR protein promotes ligand-independent survival of prostate cancer cells. Our studies support the worthiness of efforts to suppress AR’s ligand-independent function and expression of important ligand-independent AR target genes such as *c-Myc* (Figure 7). Drugs capable of suppressing AR expression are only now beginning to enter testing. These drugs include selective AR degraders and AR anti-sense oligonucleotides [37]–[40]. We await the results of these clinical studies to determine the safety, specificity, and efficacy of these agents in men with advanced prostate cancer.

## Methods

### Cell Culture

LNCaP and 22RV1 cells were purchased from American Type Culture Collection (ATCC) and grown in 10% charcoal-stripped fetal bovine serum for all experiments. Abl cells, a CRPC derivative of LNCaP, were a kind gift from Zoran Culig, PhD and were grown in RPMI with 10% charcoal-stripped fetal bovine serum. M12 prostate cancer cells expressing empty vector or AR were a kind gift from Stephen R. Plymate, PhD, and grown as described previously [9].

For RNAi experiments, LNCaP and abl cells were transfected with RNAi oligonucleotides (*AR*: 5′-GACCUACCGAGGAGCUUUCUU-3′, and *c-Myc*: 5′-GAGCUAAAACGGAGCUUUUdTdT-3′) by using DharmaFECT 3 (Dharmacon) transfection reagent for a final concentration of 50 nM [41]. 22RV1 cells were transfected with siRNA oligonucleotides by using Lipofectamine2000 (Life Technologies) transfection reagent for a final concentration of 50 nM. Cells were harvested at indicated time points post-transfection.

R1881 (Sigma) was resuspended in 100% ethanol. MDV3100 was purchased (Selleckchem) and resuspended in DMSO. JQ1 was purchased from Sigma and resuspended in DMSO. Cell growth was determined at the end of treatment with the trypan blue exclusion method (Life Technologies) following manufacturers’ instructions. All cell culture experiments were performed with biological triplicate samples and confirmed in repeat experiments.

LNCaP cells with stable overexpression of c-Myc or empty vector were generated by transfecting LNCaP cells with pCDNA-DEST40-*c-Myc* (kindly provided by Rosalie Sears, PhD) or pCMV6-AN-His (Origene) and selecting with G418.

### Colony-formation Assay

200,000 LNCaP cells with stable overexpression of empty vector (EV) or *c-Myc* were plated in 10 cm dishes. RPMI with 10% charcoal-stripped fetal bovine serum supplemented with 300 ug/ml G418 and 10 ug/ml bicalutamide treatment was added to the cells every other day for 14 days. Next, the cells were fixed with 4% formaldehyde and stained with syto60 (Invitrogen). Images were taken with Licor Odyssey imaging system.

### Immunoblotting

Immunoblotting experiments were performed as described previously [42]. Final images were obtained with Licor Odyssey imaging system. Primary antibodies used were: AR (N-20, Santa Cruz); actin (Sigma) and c-Myc (Epitomics). Secondary antibodies were purchased from Licor.

### QRT-PCR

RNA was extracted from cell pellets stored in RNAlater reagent (Life Technologies) using a MagMAX Total RNA Isolation kit (Life Technologies) or Trizol (Invitrogen) according to the manufacturer’s instructions. RNA concentration was determined using a NanoDrop ND-1000 spectrophotometer (NanoDrop). 250 ng–1 ug RNA (normalized for each experiment) was reverse-transcribed using a High-Capacity cDNA Reverse Transcription kit (Life Technologies) with random primers. Realtime (QRT)-PCR was performed using a 7500 Fast thermocycler (Life Technologies) with the following cycling program: 50°C for 2 min, 95°C for 10 min, 40 cycles of 95°C for 15 sec dissociation, 60°C for 1 min annealing/extension/read. 10 µL singleplex RTPCR reactions contained 1X Taqman universal standard mastermix, 1X Taqman hydrolysis probe, and 10 ng RNA-equivalent cDNA template. Human beta-actin (#4326315E) was used as an endogenous control. Primer information is included in Table S1. QRT-PCR for each biological replicate sample was performed with technical triplicates and analyzed with 7500 Software v2.0.5 and DataAssist Software v3.0 (Life Technologies).

### Chromatin Immunoprecipitation (ChIP)

5 µg of anti-AR antibody (N-20, Santa Cruz), anti-acetylated histone H3 (AcH3) antibody (Millipore) or normal rabbit (IgG) antibody (Millipore) were added to sheared, formaldehyde cross-linked chromatin derived from 1 to 2×10^6^ cells to immunoprecipitate DNA overnight at 4°C. 1% of chromatin was removed prior to immunoprecipitation as input. Immune complexes were collected with protein A magnetic beads (Dynabeads, Life Technologies). After extensive washing, immune complexes were released, cross-links were reversed, and DNA was purified with mini-elute PCR purification kit (Qiagen) and eluted with 60 µl EB. Realtime QPCR was performed as described above using 1X SYBR GreenER mastermix (Life Technologies), 750 ng of each primer, and 2 µl of the immunoprecipitated DNA or 2 µl of the 1% input in a 20 µL reaction. Immunoprecipitated DNA was calculated as “fold enrichment over IgG” with ddCt method. Primer information is included in Table S1.

### Statistical Analysis

Data are expressed as standard deviation of the mean. All PCR results and cell count results represent one single experiment performed in triplicate. p-values were calculated from one single experiment with two-tailed unpaired student’s T-test in Excel (Microsoft) or Study Results 1.0 software. Each experiment was confirmed in two to three separate experiments.

### AR ChIP-microarray Data Mining

The AR ChIP-microarray data was downloaded from http://research4.dfci.harvard.edu/brownlab/datasets/index.php?dir=Wang_AR_Data/for both abl and LNCaP cells. We filtered the data requiring that each peak have a FDR <5% and be within 50 Kb of either the 5′ or 3′ end of a RefSeq gene. Using these criteria, we found overlapping peaks with proximity to the *c-Myc* locus at chr8∶128844491–128845592 (∼21.6 Kb from *c-Myc*) in abl cells and chr8∶128844572–128845592 (∼21.7 Kb from *c-Myc*) in LNCaP cells.

### *AR* and *c-Myc* Expression in Human Castration-Resistant Prostate Cancer Tumors

Agilent 44 K whole human genome expression oligonucleotide microarrays (Agilent Technologies, Inc.) were used to profile 140 human castration-resistant prostate cancer soft tissue metastases from 55 patients. Tissue samples were collected from autopsies performed at the University of Washington Medical Center under the rapid autopsy program with Institutional Review Board approval as described previously [43]. The tumor samples were laser-capture microdissected, and total RNA was isolated and amplified as described previously [44]. Probe labeling and hybridization were performed following the Agilent suggested protocols, and fluorescent array images were collected using the Agilent DNA microarray scanner G2565BA. Expression ratios were normalized using the R software Bioconductor snm package and combined with expression profiles from 15 normal prostate specimens to create z-scores, which represent the number of standard deviations away from the mean of expression in the normal prostate group [45], [46]. GraphPad Prism v4.03 software was used to analyze the correlation of expression and strength of association between genes. A Pearson correlation coefficient, linear regression, and F test for significantly non-zero slope were performed for each pair of genes as well as a Fisher’s exact test and odds ratio on the contingency table analyzing the co-occurrence of tumors with *AR* or *c-Myc* z-scores greater than 2.

## Supporting Information

Figure S1 **Co-suppression of** ***AR*** **and** ***c-Myc*** **does not lead to greater anti-tumor activity than suppression of either protein by itself.** A) LNCaP cells were transfected with 50 nM of NTC, *AR*, *c-Myc*, or both *AR* and *c-Myc* RNAi oligonucleotides. Cells were switched to charcoal-stripped serum on the day of transfection. Cell growth was determined 5 days later with the trypan blue exclusion method. *denotes p<0.01 compared to NTC. B) Immunoblotting was performed to determine the levels of AR, c-Myc, and actin.
(TIF)

Figure S2 ***c-Myc*** **over expression promotes ligand-independent prostate cancer growth.** A) The same number of LNCaP cells with stable overexpression of empty vector or c-Myc were grown in 10% charcoal-stripped fetal bovine serum. Cell number was determined 1, 4, and 7 days after plating with the trypan blue exclusion method. Cell growth was calculated compared to day 1. p<0.01 for both time points. B) The same number of LNCaP cells with stable overexpression of empty vector or c-Myc was plated. Cells were grown in charcoal-stripped serum supplemented with bicalutamide for 14 days. Colony formation was determined.
(TIF)

Figure S3 **MDV3100 treatment does not reduce** ***c-Myc*** **expression.** LNCaP, abl, and 22RV1 cells were grown in androgen-replete serum and treated with 10 µM MDV3100 or vehicle for 24 hours. QRT-PCR was performed to determine the mRNA levels of *KLK3* and *c-Myc* relative to *actin*. *denotes p<0.001 compared to vehicle.
(TIF)

Figure S4 **AR overexpression promotes** ***c-Myc*** **upregulation.** AR or empty vector was stably overexpressed in M12 prostate cancer cells that do not express endogenous AR [9]. RNA and protein were harvested. A) QRT-PCR was performed to determine the level of *c-Myc* relative to *actin*. B) Immunoblotting was performed to determine the levels of AR, c-Myc and actin. *denotes p<0.003 compared to empty vector.
(TIF)

Table S1 **Primers used for ChIP-PCR and QRTPCR.**
(DOCX)
