# Supplementary figures and images for: Androgen Receptor Promotes Ligand-Independent Prostate Cancer Progression through c-Myc Upregulation
Source: PLoS One. 2013 May 21;8(5):e63563. doi: 10.1371/journal.pone.0063563 (PMC3660401; doi:10.1371/journal.pone.0063563)

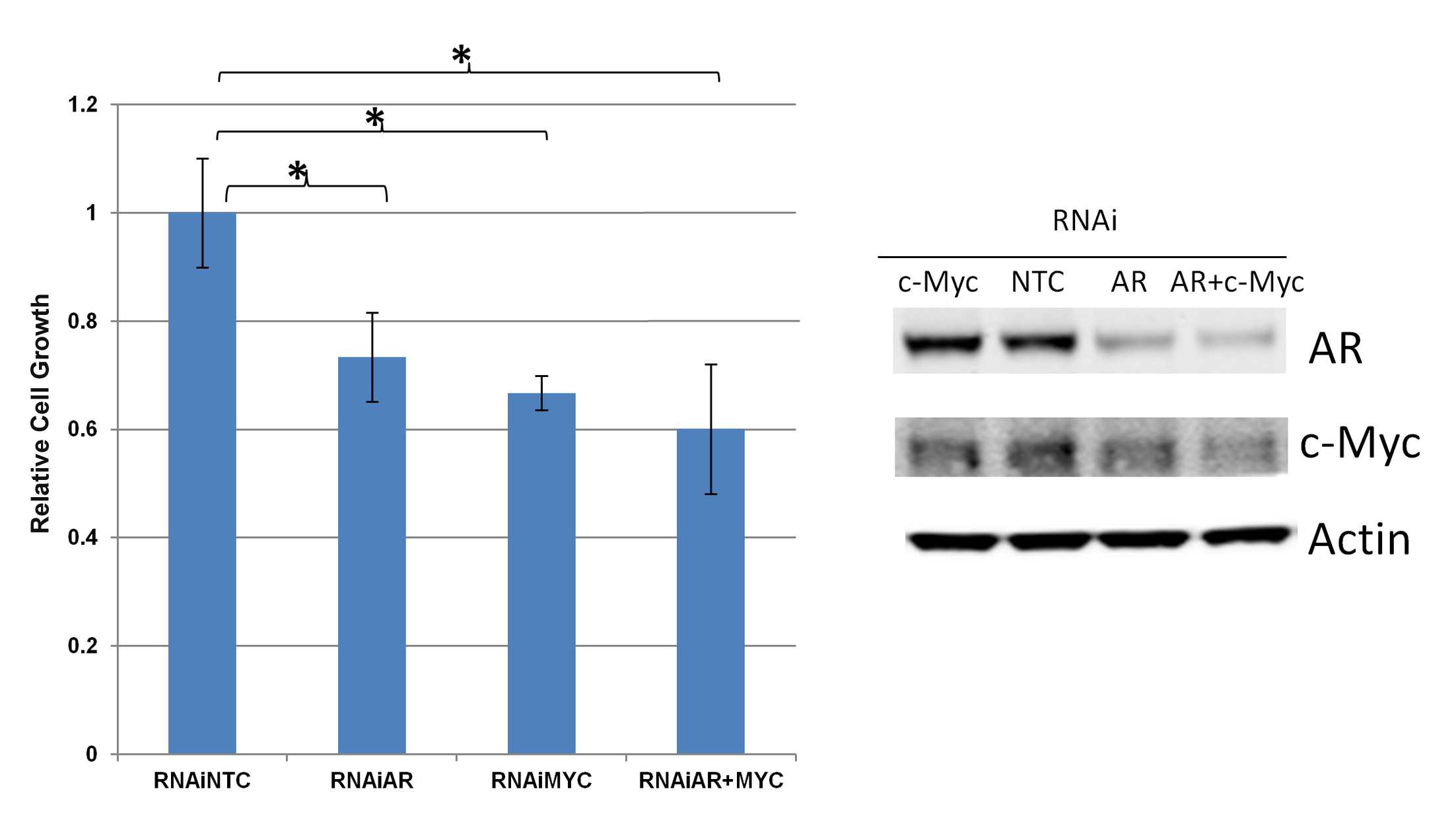

Supplement: Figure S1 — Co-suppression of AR and c-Myc does not lead to greater anti-tumor activity than suppression of either protein by itself. A) LNCaP cells were transfected with 50 nM of NTC, AR, c-Myc, or both AR and c-Myc RNAi oligonucleotides. Cells were switched to charcoal-stripped serum on the day of transfection. Cell growth was determined 5 days later with the trypan blue exclusion method. *denotes p<0.01 compared to NTC. B) Immunoblotting was performed to determine the levels of AR, c-Myc, and actin. (TIF) [file pone.0063563.s001.tif]

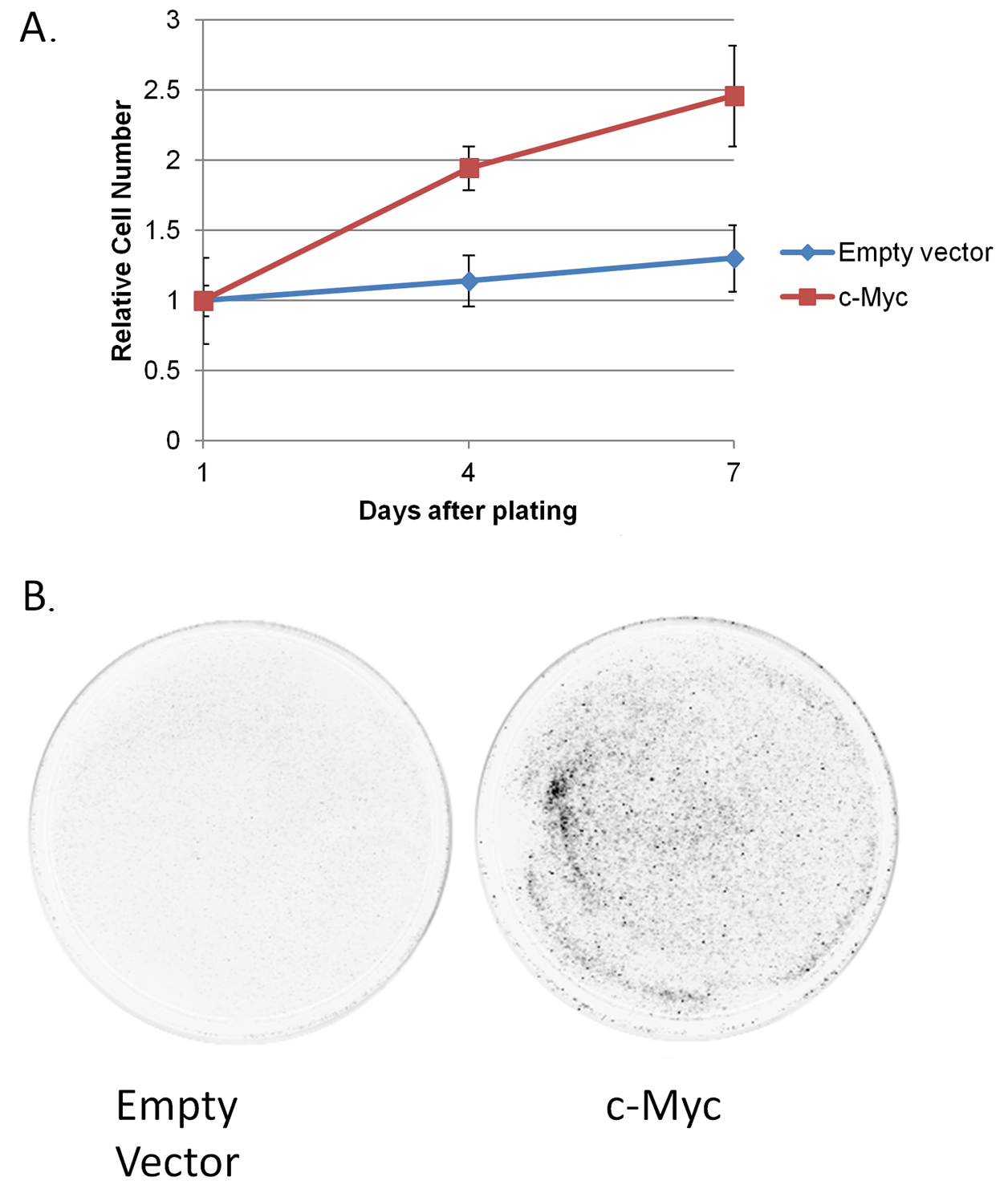

Supplement: Figure S2 — c-Myc over expression promotes ligand-independent prostate cancer growth. A) The same number of LNCaP cells with stable overexpression of empty vector or c-Myc were grown in 10% charcoal-stripped fetal bovine serum. Cell number was determined 1, 4, and 7 days after plating with the trypan blue exclusion method. Cell growth was calculated compared to day 1. p<0.01 for both time points. B) The same number of LNCaP cells with stable overexpression of empty vector or c-Myc was plated. Cells were grown in charcoal-stripped serum supplemented with bicalutamide for 14 days. Colony formation was determined. (TIF) [file pone.0063563.s002.tif]

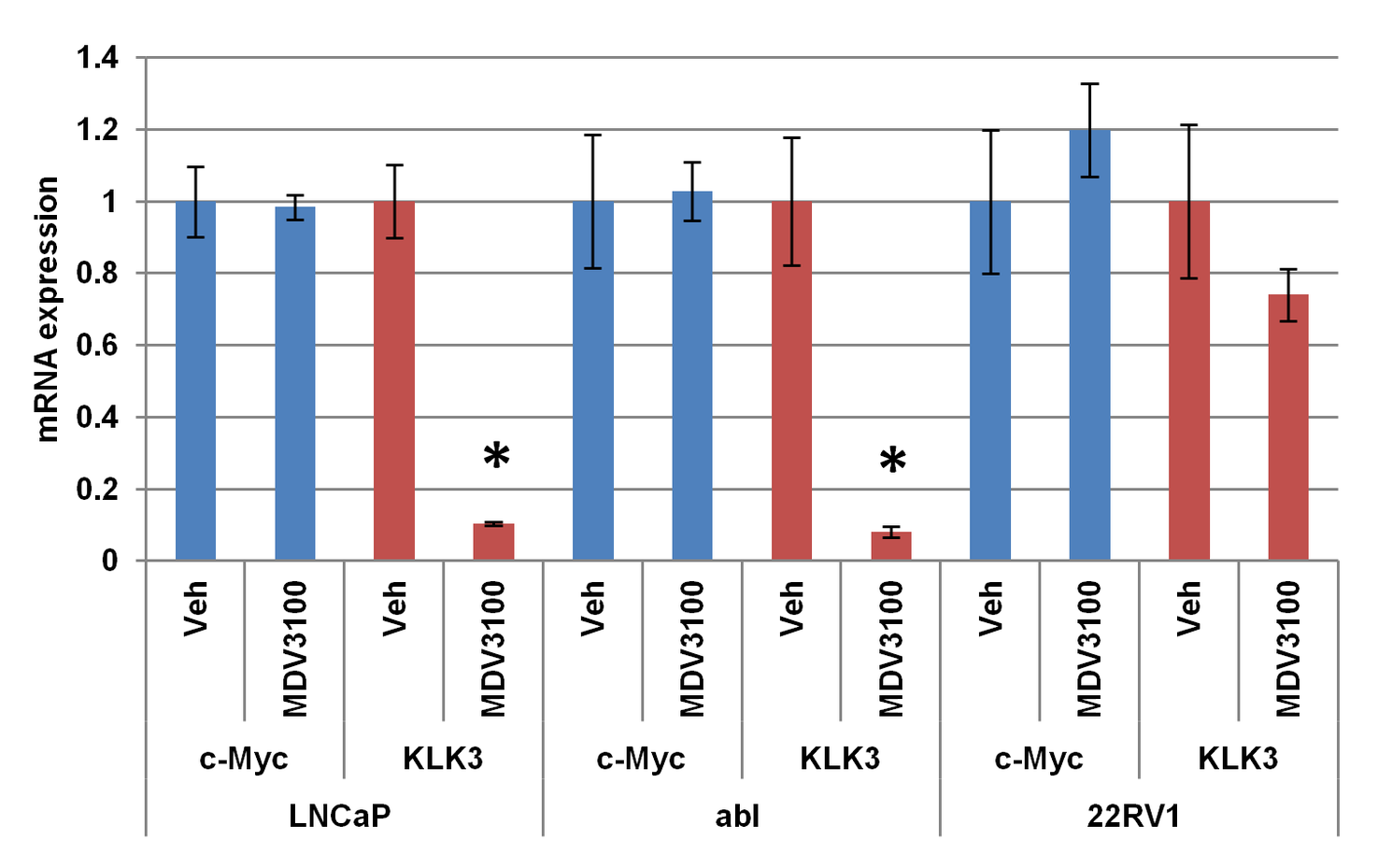

Supplement: Figure S3 — MDV3100 treatment does not reduce c-Myc expression. LNCaP, abl, and 22RV1 cells were grown in androgen-replete serum and treated with 10 µM MDV3100 or vehicle for 24 hours. QRT-PCR was performed to determine the mRNA levels of KLK3 and c-Myc relative to actin. *denotes p<0.001 compared to vehicle. (TIF) [file pone.0063563.s003.tif]

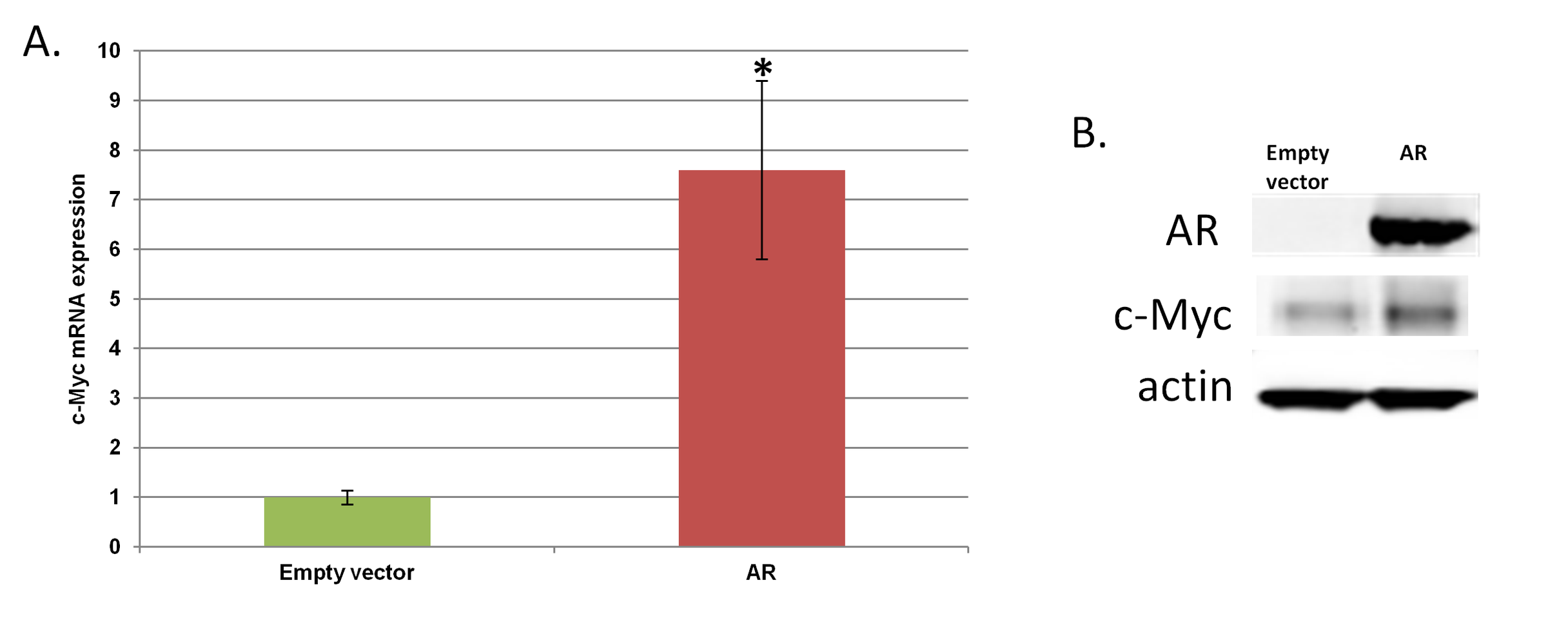

Supplement: Figure S4 — AR overexpression promotes c-Myc upregulation. AR or empty vector was stably overexpressed in M12 prostate cancer cells that do not express endogenous AR [9]. RNA and protein were harvested. A) QRT-PCR was performed to determine the level of c-Myc relative to actin. B) Immunoblotting was performed to determine the levels of AR, c-Myc and actin. *denotes p<0.003 compared to empty vector. (TIF) [file pone.0063563.s004.tif]
