# Supplementary material for: Androgen Receptor Promotes Ligand-Independent Prostate Cancer Progression through c-Myc Upregulation
Source: PLoS One. 2013 May 21;8(5):e63563. doi: 10.1371/journal.pone.0063563 (PMC3660401; doi:10.1371/journal.pone.0063563)
Supplement: Table S1 — Primers used for ChIP-PCR and QRTPCR. (DOCX) [file pone.0063563.s005.docx]

**Supplementary Table 1: Primers used for ChIP-PCR and QRTPCR**

| *KLK3* enhancer sense ChIP primer | TGGGACAACTTGCAAACCTG |
| --- | --- |
| *KLK3* enhancer anti-sense ChIP primer | CCAGAGTAGGTCTGTTTTCAATCCA |
| *c-Myc* enhancer sense ChIP primer | CCAGCGAATTATTCAGAA |
| *c-Myc* enhancer anti-sense ChIP primer | AATTACCATTGACTTCCTC |
| *c-Myc* Taqman assay | Hs00905030_m1 |
| *AURKB* Taqman assay | Hs00177782_m1 |
| *E2F1* Taqman assay | Hs00153451_m1 |
| *KIF11* Taqman assay | Hs00189698_m1 |
| *CDKN1A* Taqman assay | Hs00355782_m1 |
| *TPX2* Taqman assay | Hs00201616_m1 |
